# Multifocal Acquired Demyelinating Sensory and Motor Neuropathy: An Atypical Chronic Inflammatory Demyelinating Polyradiculoneuropathy Variant Responsive to Intravenous Immunoglobulin

**DOI:** 10.7759/cureus.115924

**Published:** 2026-09-07

**Authors:** Ananya Sukhija, Lyubov P Smertina

**Affiliations:** 1 Medicine, Surgut State University, Surgut, RUS; 2 Neurology, Surgut State University, Surgut, RUS

**Keywords:** chronic inflammatory demyelinating polyneuropathy, conduction block, demyelinating neuropathy, intravenous immunoglobulin, lewis-sumner syndrome, madsam

## Abstract

We report the case of a male patient in his mid-30s with a progressive, fluctuating sensorimotor neuropathy beginning in 2022. His symptoms were marked by asymmetric weakness, predominantly affecting the lower limbs, along with distal sensory disturbances, including numbness and paraesthesia. Functional impairment progressed over time, significantly affecting daily activities.

Electrophysiological studies revealed a generalized, asymmetric, multifocal demyelinating neuropathy with conduction blocks at atypical, non-compressive sites. These findings were consistent with multifocal acquired demyelinating sensory and motor neuropathy (MADSAM). The patient showed no clinical response to corticosteroid therapy but achieved significant improvement following intravenous immunoglobulin (IVIG) treatment.

## Introduction

Chronic inflammatory demyelinating polyradiculoneuropathy (CIDP) is an acquired immune-mediated neuropathy causing progressive or relapsing motor and sensory dysfunction associated with peripheral nerve demyelination [1]. Typical CIDP presents with symmetrical weakness and sensory deficits. However, several CIDP variants have been recognized, reflecting the clinical heterogeneity of the disease [2-4].

Multifocal acquired demyelinating sensory and motor neuropathy (MADSAM), also known as Lewis-Sumner syndrome, is a multifocal CIDP variant characterized by asymmetric, multifocal involvement of peripheral nerves [3,4]. Patients commonly present with focal weakness and sensory disturbances, often mimicking other neuropathies and leading to diagnostic delay.

Electrophysiological studies are central to diagnosis, particularly through the identification of demyelinating abnormalities such as conduction block and temporal dispersion in appropriate clinical contexts. The 2021 European Academy of Neurology/Peripheral Nerve Society (EAN/PNS) guideline emphasizes the integration of the clinical phenotype, electrophysiological evidence of demyelination, and exclusion of alternative diagnoses when evaluating CIDP and its variants [4]. Treatment options include intravenous immunoglobulin (IVIG), corticosteroids, and plasma exchange, with IVIG or corticosteroids recommended as initial therapies for CIDP variants [4].

This report describes a patient with MADSAM/multifocal CIDP characterized by progressive asymmetric sensorimotor involvement, electrophysiological evidence of multifocal demyelination, delayed diagnostic recognition, and reported clinical improvement following IVIG therapy. The case highlights the diagnostic challenge of recognizing multifocal CIDP in a patient with an asymmetric and fluctuating clinical course.

## Case presentation

Clinical summary

A male patient in his mid-30s with no significant past medical or family history developed a progressive and fluctuating sensorimotor neuropathy beginning in 2022, initially characterized by predominantly lower-limb weakness and subsequently progressing to asymmetric involvement of both lower and upper limbs.

The symptoms developed insidiously, with gradual onset of weakness predominantly affecting the lower limbs, accompanied by a wide-based gait and impaired balance. The weakness progressively worsened and became asymmetric, with greater involvement of the right side than the left. Upper-limb involvement subsequently developed, resulting in progressive impairment of fine motor function.

Neurological examination

As the disease progressed, the patient developed increasing difficulty with ambulation and at times required physical support for walking. During exacerbations, he was unable to climb stairs. Upper-limb involvement resulted in impaired fine motor function, including difficulty with tasks such as buttoning clothes and tying shoelaces.

Sensory symptoms included numbness and tingling, with reduced vibration sensation and no associated neuropathic pain or dysesthesia. The available clinical documentation did not provide a complete quantitative description of the sensory distribution.

No autonomic symptoms, including bladder or bowel dysfunction, were reported. The clinical course was fluctuating, with periods of partial improvement followed by recurrent worsening of motor and functional symptoms.

Clinical investigations and treatment

The patient first sought medical evaluation in 2025 and received a diagnosis in November of the same year. Electrophysiological studies using electroneuromyography (ENMG) demonstrated a generalized, asymmetric, multifocal neuropathy with predominantly motor involvement and demyelinating features. Representative motor nerve conduction studies of the bilateral lower extremities are shown in Figure 1, while representative sensory nerve conduction and F-wave studies are shown in Figure 2. The complete ENMG examination included additional motor and sensory studies of the upper and lower extremities. Representative quantitative abnormalities from the complete examination are summarized in Table 1.

**Figure 1:**
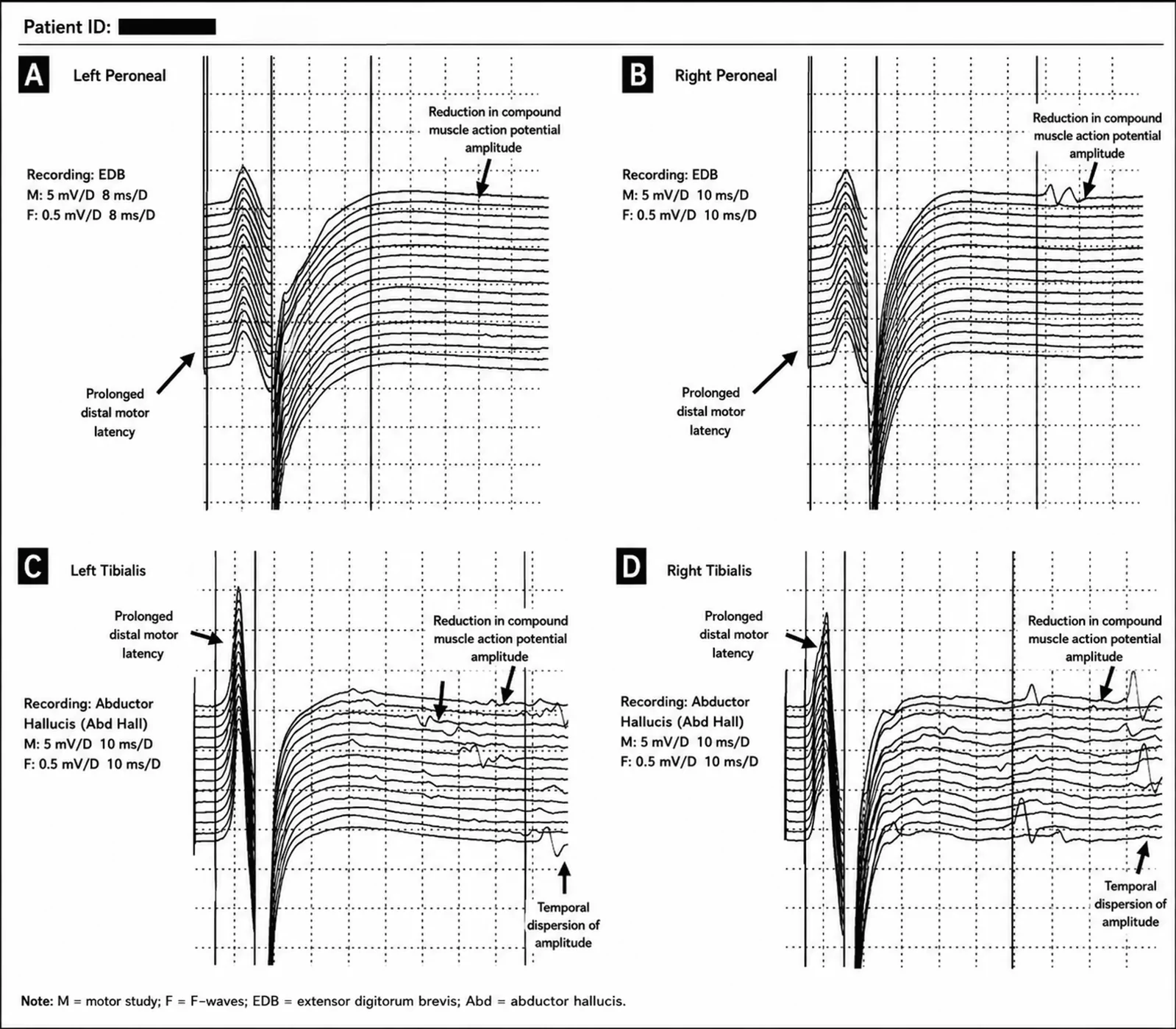
Motor nerve conduction studies of the bilateral lower extremities. (A) Left peroneal nerve recording from the EDB. (B) Right peroneal nerve recording from the EDB. (C) Left tibial nerve recording from the abductor hallucis (AH). (D) Right tibial nerve recording from the AH.

**Figure 2:**
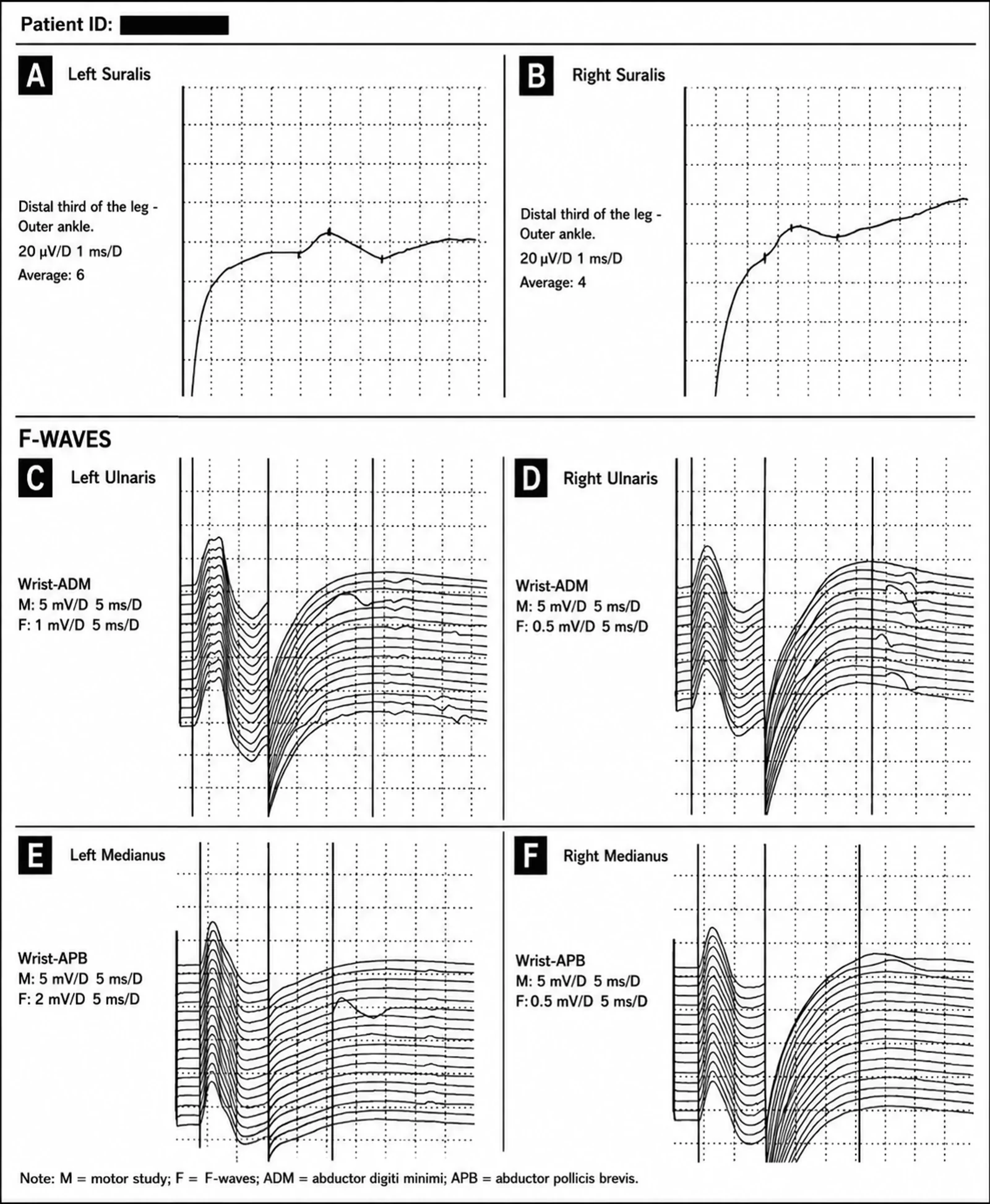
Sensory nerve conduction studies and F-wave analyses. (A) Left sural sensory nerve action potential (SNAP). (B) Right SNAP. (C) Left ulnar nerve F-wave recordings recorded from the ADM. (D) Right ulnar nerve F-wave recordings recorded from the ADM. (E) Left median nerve F-wave recordings recorded from the APB. (F) Right median nerve F-wave recordings recorded from the APB.

**Table 1:** Summary of quantitative electrophysiological abnormalities. CMAP: compound muscle action potential; SNAP: sensory nerve action potential

Nerve	Study	Key quantitative finding	Interpretation
Left ulnar	Motor	CMAP amplitude reduction up to 70%; conduction velocity 44-56 m/s; F-wave minimum latency 33.1 ms	Motor conduction block with demyelinating features
Right ulnar	Motor	CMAP amplitude reduction up to 82%; conduction velocity 44-63 m/s; F-wave minimum latency 32.7 ms	Motor conduction block with demyelinating features
Left median	Motor	CMAP amplitude reduction up to 34%; conduction velocity 53-62 m/s; F-wave loss 90%; F-wave minimum latency 26.0 ms	Multifocal motor conduction abnormality
Right median	Motor	CMAP amplitude reduction up to 85%; conduction velocity 53-54 m/s; F-wave loss 80%; F-wave minimum latency 30.7 ms	Multifocal motor conduction abnormality
Left radial	Motor	CMAP amplitude reduction up to 53%; conduction velocity 51-66 m/s	Multifocal motor conduction abnormality
Right radial	Motor	CMAP amplitude reduction up to 50%; conduction velocity 64-69 m/s	Multifocal motor conduction abnormality
Left peroneal	Motor	CMAP amplitude reduction 16%; conduction velocity 44-46 m/s	Motor conduction abnormality
Right peroneal	Motor	CMAP amplitude reduction 29%; conduction velocity 42 m/s; F-wave loss 90%; F-wave minimum latency 68.9 ms	Motor conduction abnormality
Left tibial	Motor	CMAP amplitude reduction 60%; conduction velocity 45 m/s	Motor conduction block
Right tibial	Motor	CMAP amplitude reduction 59%; conduction velocity 44 m/s	Motor conduction block
Left sural	Sensory	SNAP amplitude 12.2 mV; conduction velocity 44 m/s	Sensory conduction documented
Right sural	Sensory	SNAP amplitude 11.3 mV; conduction velocity 48 m/s	Sensory conduction documented

The complete ENMG examination demonstrated multifocal abnormalities involving multiple motor and sensory nerves. Representative findings from selected lower-limb motor studies and upper-limb sensory/F-wave studies are shown in Figures 1-2, respectively. The principal electrophysiological abnormalities documented across the complete examination are summarized in Table 1.

The patient's clinical course, diagnostic evaluation, and treatment milestones are summarized in Table 2.

**Table 2:** Clinical timeline of disease progression, diagnostic evaluation, and treatment. ENMG: electroneuromyography; MADSAM: multifocal acquired demyelinating sensory and motor neuropathy; CIDP: chronic inflammatory demyelinating polyradiculoneuropathy; IVIG: intravenous immunoglobulin

Year	Event
2022	Initial onset of progressive lower-limb weakness with gait and balance impairment
2023	Progressive worsening of lower-limb weakness and functional mobility
2024	Development of upper-limb involvement, asymmetric weakness, and impaired fine motor function
2025	Neurological evaluation and ENMG demonstrating multifocal demyelinating abnormalities; diagnosis of MADSAM/multifocal CIDP established
2025	Corticosteroid therapy administered without sustained clinical improvement; IVIG therapy initiated, followed by reported clinical improvement

This case highlights the diagnostic challenges associated with atypical CIDP variants and emphasizes the importance of electrophysiological evaluation in identifying multifocal demyelinating patterns. It also underscores variability in treatment response, particularly steroid resistance with a favorable response to IVIG.

Cerebrospinal fluid (CSF) analysis revealed clear fluid with a cytosis of 1 cell/µL and a total protein concentration of 0.28 g/L. The Pandy reaction was positive (++), while chloride and glucose concentrations were 120 mmol/L and 4.6 mmol/L, respectively. Although the total CSF protein concentration was within the reported laboratory reference range, the positive Pandy reaction represented a qualitative protein/globulin precipitation finding and was considered nonspecific; it did not independently establish albuminocytologic dissociation. The CSF laboratory findings are summarized in Table 3.

**Table 3:** Cerebrospinal fluid laboratory findings.

Parameter	Patient result	Unit	Reference range
Appearance	Clear/transparent	-	Clear/colorless
Cytosis (white blood cell count)	1	cells/µL	0-5
Protein	0.28	g/L	0.15-0.60
Pandy reaction	++	-	Negative
Chloride	120	mmol/L	115-130
Glucose	4.6	mmol/L	2.77-4.44

The patient received supportive therapy, including intramuscular B vitamins (thiamine and pyridoxine, 50 mg administered on alternate days) and gastroprotective treatment with omeprazole 20 mg once daily. Corticosteroid therapy with intravenous dexamethasone (8 mg twice daily) was initiated; however, no sustained clinical improvement was observed. The patient was subsequently treated with IVIG (Immunovenin®, 1.25 g per vial, 50 mg/mL), resulting in significant clinical improvement.

## Discussion

CIDP encompasses a spectrum of immune-mediated neuropathies with diverse clinical presentations. Among its atypical variants, MADSAM, also known as Lewis-Sumner syndrome, is characterized by asymmetric, multifocal involvement of peripheral nerves and is frequently associated with conduction block on electrophysiological studies [2,3].

Our patient displayed the classical hallmarks of MADSAM, including progressive asymmetric weakness, multifocal neurological involvement, and conduction blocks occurring at non-compressive sites. These findings are important in differentiating MADSAM from other peripheral neuropathies, particularly those with predominantly symmetrical or axonal patterns. The electrophysiological findings were consistent with a demyelinating neuropathy according to European Federation of Neurological Societies/PNS (EFNS/PNS) criteria.

Electrophysiological evaluation played a central role in establishing the diagnosis. The finding of multifocal conduction blocks and temporal dispersion strongly supported an immune-mediated demyelinating process rather than compressive, hereditary, or degenerative etiologies. This clinical picture underscores the importance of comprehensive nerve conduction studies in patients presenting with atypical or asymmetric neuropathic symptoms.

An important aspect of this case is the variability in treatment response. Although corticosteroids are commonly used in the management of CIDP, the absence of clinical improvement following steroid therapy underscores the heterogeneity of treatment response observed in atypical CIDP variants. In contrast, the patient demonstrated significant clinical improvement following IVIG therapy, consistent with current evidence supporting its efficacy in immune-mediated demyelinating neuropathies [4].

Previous studies have demonstrated considerable variability in the clinical course and treatment response of MADSAM. In a study of 23 patients with Lewis-Sumner syndrome, the disease course was chronic progressive in 71% of patients and relapsing-remitting in the remainder. During follow-up, approximately half of the patients continued to demonstrate a multifocal pattern, with the distribution of motor deficits remaining similar to the initial presentation [5]. Similarly, a retrospective study of 15 patients found that IVIG was effective in seven patients after one year, while six remained stable and two worsened. Two patients experienced marked worsening following corticosteroid treatment [6]. These findings are consistent with the present case, in which the patient had a progressive but fluctuating course, showed no sustained clinical improvement with corticosteroid therapy, and subsequently demonstrated significant improvement following IVIG. However, corticosteroid resistance is not universal in MADSAM, highlighting the heterogeneous therapeutic response of this disorder [5,6]. Current EAN/PNS guidelines recommend IVIG or corticosteroids as initial treatment options for CIDP and its variants, with plasma exchange recommended when IVIG and corticosteroids are ineffective [4]. Thus, the favorable response to IVIG observed in this patient is consistent with the existing literature, while the lack of response to corticosteroids represents a clinically relevant feature of the individual disease course.

Interestingly, CSF analysis revealed a protein level of 0.28 g/L without pleocytosis. Although albuminocytologic dissociation is a classical feature of CIDP, normal CSF findings do not exclude the diagnosis and have been reported in atypical CIDP variants, including MADSAM. This emphasizes the importance of integrating clinical and electrophysiological findings rather than relying on a single diagnostic modality.

The three-year gap between symptom onset (2022) and evaluation (2025) likely contributed to disease progression and functional impairment in this patient. Earlier recognition of asymmetric neuropathic symptoms and timely electrophysiological assessment may facilitate earlier diagnosis and initiation of effective immunomodulatory therapy.

Overall, this case highlights the diagnostic complexity of MADSAM and reinforces the importance of combining clinical evaluation, electrophysiological evidence, and therapeutic response when assessing atypical CIDP variants. Recognition of steroid-resistant disease with a favorable response to IVIG may help guide management and improve outcomes in similar patients.

## Conclusions

MADSAM is an atypical variant of CIDP characterized by asymmetric clinical involvement and multifocal conduction block. This case highlights the importance of comprehensive electrophysiological evaluation in patients with non-classical neuropathic presentations to facilitate accurate diagnosis.

Our case also demonstrates variability in therapeutic response, with no sustained clinical improvement documented during corticosteroid therapy and clinical improvement reported following IVIG treatment. Early recognition of characteristic clinical and electrophysiological features may facilitate timely diagnostic evaluation and initiation of appropriate immunomodulatory treatment.
